# Patterned expression of neurotrophic factors and receptors in human limbal and corneal regions

**Published:** 2007-10-16

**Authors:** Hong Qi, Eliseu Yung Chuang, Kyung-Chul Yoon, Cintia S. de Paiva, H. David Shine, Dan B. Jones, Stephen C. Pflugfelder, De-Quan Li

**Affiliations:** 1 Ocular Surface Center, Cullen Eye Institute, Department of Ophthalmology and; 2 Center for Cell and Gene Therapy and Department of Neurosurgery, Baylor College of Medicine, Houston, TX;; 3 Peking University Eye Center, Peking University Third Hospital, Beijing, China;; 4 Department of Ophthalmology, Chonnam National University Medical School and Hospital, Gwang-Ju, Republic of Korea

## Abstract

**Purpose:**

To evaluate the expression patterns of neurotrophic factors (NTFs) and their receptors in the human cornea with the intention of exploring the role of NTFs in maintaining corneal epithelial stem cells in the limbus.

**Methods:**

Fresh human corneoscleral tissues were prepared for frozen sections. Immunofluorescent staining was performed with primary antibodies against six members of three NTF families and their six receptors. To confirm the specificity of NTF primary antibodies, neutralization experiments with their corresponding peptides and western blot analysis were performed.

**Results:**

Based on spatial and differential immuno-localization, three patterns of NTF expression were potentially involved in epithelial-mesenchymal interaction on the ocular surface: (1) the epithelial type: nerve growth factor (NGF) and glial cell-derived neurotrophic factor (GDNF); (2) the paracrine type: neurotrophin (NT)-3 and NT-4/5; and (3) the reciprocal type: brain-derived neurotrophic factor (BDNF). The stem cell-enriched basal cells of the limbal epithelium expressed three unique staining patterns for NTFs: (1) exclusively positive for NGF, GDNF, and their corresponding receptors, TrkA and GDNF family receptor alpha (GFRα)-1; (2) relatively high levels of BDNF; and (3) negative for NT-3 and NT-4. Additionally, the neurotrophin common low-affinity receptor, p75NTR, was mainly expressed by the basal layer of the entire corneal and limbal epithelia, and TrkB and TrkC were evenly expressed by the entire corneal and limbal epithelia. BDNF, p75NTR, TrkB, and TrkC are also abundantly expressed by limbal stroma cells. No specific immunoreactivity to ciliary neurotrophic factor (CNTF) and its receptor, CNTFRα, was detected in cornea tissue in situ.

**Conclusions:**

Our findings revealed patterned expression of NTFs and their receptors in the human ocular surface, suggesting that they may play a vital role in maintaining corneal epithelial stem cells in the limbus. NGF, GDNF, GFRα-1, TrkA, and BDNF may serve as new limbal basal cell markers defining the corneal epithelial stem cell phenotype.

## Introduction

Neurotrophic factors (NTFs) are a family of polypeptides that are derived from the neuron's target cells and promote survival of peripheral and central neurons by protecting them from apoptosis. The neurotrophins are the best characterized family of neurotrophic factors that comprises nerve growth factor (NGF), brain-derived neurotrophic factor (BDNF), neurotrophin (NT)-3, NT-4/5, and NT-6 (see review articles [1,2]). They share the same low-affinity neurotrophin receptor, p75NTR, but use different members of the Trk receptor tyrosine kinase family for high-affinity binding and signal transduction. NGF preferentially activates TrkA; BDNF and NT-4/5 preferentially bind to TrkB; and NT-3 signals through TrkC. Other major NTF families include the glial cell line-derived neurotrophic factor (GDNF) family and the ciliary neurotrophic factor (CNTF) family. GDNF and its related family members, neurturin, artemin, and persephin, signal through the same high-affinity receptor, Ret receptor tyrosine kinase. The ligand specificity of GDNF is determined by a novel class of glycosylphosphatidylinositol (GPI)-anchored proteins called the GDNF family receptor alpha (GFRα) 1–4. GDNF preferentially binds to GFRα1 [3]. CNTF differs distinctly from other neurotrophic molecules (i.e., NGF, BDNF, and NT-3) in both its molecular characteristics (CNTF is a cytosolic rather than a secretory molecule) and its broad spectrum of biologic activities [4,5]. The receptor for CNTF consists of a ligand-binding subunit, CNTFRα, and two α subunits, gp130 and leukemia inhibitory factor (LIF) receptor α [4].

It is well known that corneal epithelial homeostasis is governed by a small subpopulation of corneal epithelial stem cells (SCs) located in the basal epithelial layer of the limbus [6-8]. We have previously reported three types of cytokines and growth factors participating in epithelial-mesenchymal interaction on the human ocular surface and the difference of their expression patterns between the corneal and limbal regions [9]. This cytokine network plays an important role in regulating proliferation and differentiation of the corneal epithelium. Traditionally, NTFs are defined as target-derived, anti-apoptotic molecules that maintain embryonic or adult neuronal cells. NTFs regulate survival and differentiation of neurons and neural stem cells throughout the nervous system during embryonic and postnatal development [10-14]. However, although NFs are defined as molecules that maintain neuronal cells, studies have shown that NTFs possess a diverse range of biologic effects in non-neuron cells and can act in an autocrine or paracrine fashion on stem cells outside the nervous system (see review [15]). For example, neurotrophin signaling was found to promote survival of embryo stem cells [16], oocytes [17], and esophageal and oral keratinocyte stem cells [18,19]. BDNF is expressed in skeletal muscle satellite cells and plays a key role in maintaining the population of adult muscle progenitors [20]. GDNF was identified as the essential growth factor supporting self-renewal of spermatogonial stem cells in vitro [21,22]. GDNF receptor, GFRα-1, is strongly expressed by a subset of spermatogonia including the stem cells for spermatogenesis [23,24].

The expression and function of NTFs in the ocular surface epithelia has also been investigated. BDNF and NT-3 have been found in the corneal epithelium of the mature mouse eye [25]. Transcripts encoding NGF, BDNF, NT-3, and receptors Trk A, Trk B, Trk C, GFRα-1 were detected in the corneal epithelium and/or stroma ex vivo as well as in cultured corneal epithelium [26,27]. NGF and GDNF have been shown to stimulate epithelial migration, colony formation, and proliferation whereas BDNF only enhanced colony formation [26,27]. TrkA, the high affinity NGF receptor, has been shown to preferentially localize to limbal basal epithelial cells [28]. The clinical significance of these factors was shown by demonstrating that topical NGF treatment of patients with neurotrophic corneal ulcers promoted epithelial and stromal healing and restored corneal integrity [29]. Despite these findings, the spatial expression and localization of these NTFs and their role in the stem cell-containing limbus remains undefined. The purpose of this study was to evaluate the expression patterns of six important NTFs and their receptors in human corneal and limbal tissues with the intention of exploring the potential role of these NTFs in maintaining corneal epithelial stem cells in the limbal niche.

## Methods

### Materials and reagents

Optimal cutting temperature (OCT) compound and cryomolds were from Sakura Finetek (Torrance, CA). Affinity-purified rabbit polyclonal antibodies (pAb) against NGF (M-20), BDNF (N-20), NT-3 (N-20), NT-4 (N-20), TrkA (763), TrkB (H-181), TrkC (798), GFRα-1 (H-70); goat pAb against CNTF (R-20); and mouse monoclonal antibody (mAb) against CNTF receptor, CNTFRα (AN-B2), all corresponding blocking peptides for NGF, BDNF, TrkA, and GFRα-1 specific antibodies, were purchased from Santa Cruz Biotechnology (Santa Cruz, CA). Affinity-purified mAb against p75NTR (ME20.4) were purchased from Chemicon International (Temecula, CA). Affinity-purified rabbit pAb against GDNF was purchased from Laboratory Vision (Fremont, CA). Anti β-actin rabbit pAb was from Cell Signaling Technology Inc. (Danvers, MA). Fluorescein Alexa Fluor 488 (green) or 594 (red) conjugated goat anti-rabbit or anti-mouse IgG, donkey anti-goat IgG, and 0.25% trypsin/0.03% EDTA solution were from Invitrogen (Carlsbad, CA). Propidium iodide (PI) and other chemicals were purchased from Sigma-Aldrich (St. Louis, MO). Protease inhibitor cocktail tablets were from Roche Applied Science (Indianapolis, IN). Horseradish peroxidase (HRP)-conjugated goat anti-rabbit IgG, goat anti-mouse IgG, and the BCA protein assay kit were from Pierce Chemical (Rockford, IL). Enhanced Chemiluminescence (ECL) reagents were from Amersham Biosciences (Piscataway, NJ). Ready Gel for protein electrophoresis (4%–15% Tris-HCl), sodium dodecyl sulfate (SDS), prestained SDS–PAGE low range standards, precision plus protein standards, precision protein strep tactin-HRP conjugate were from Bio-Rad (Hercules, CA). Immobilon-P polyvinylidendifluoride (PVDF) membrane was from Millipore (Billerica, MA).

### Immunofluorescent staining

Fresh normal human corneal tissues (less than 48 h postmortem) were obtained for immunostaining from the National Disease Research Interchange (NDRI, Philadelphia, PA). The corneal and limbal specimens were prepared using a previously described method [30] by cutting the tissues in the vertical meridian from 6 to 12 o'clock through the central cornea and in the horizontal direction across the superior peripheral cornea and limbus. The tissue specimens were embedded in a mixture of 75% OCT compound (Sakura Finetek, Inc.) and 25% Immu-Mount (v/v; Thermo-Shandon, Pittsburgh, PA), frozen in liquid nitrogen, and cut into frozen sections (10 μm thick) for immunostaining.

Immunofluorescent staining was performed to evaluate expression and location of six NTFs and six receptors in human corneal and limbal frozen sections, using a previously reported method [9,30,31]. In brief, human corneal and limbal frozen sections were thawed, dehydrated, and fixed in cold methanol: acetone (1:1) at −30 °C for 3 min. Sections were blocked with 20% normal goat or donkey serum in phosphate buffered saline (PBS) for 1 h to reduce nonspecific antibody interaction. Primary antibodies against human NGF (1:200), BDNF (1:100), NT3 (1:100), NT-4 (1:100), GDNF (1:100), CNTF (1:100), TrkA (1:200), TrkB (1:100), Trk C (1:100), p75NTR (1:100), GFRα-1 (1:50), and CNTFRα (1:100) were applied and incubated for 2 h at room temperature (RT). Secondary antibodies, Alexa-Fluor 488 conjugated goat anti-rabbit or anti-mouse IgG, or donkey anti-goat IgG (1:300) were then applied and incubated in a dark chamber for 1 h followed by counterstaining with propidium iodide (1:200) for 5 min. After washing with PBS, antifade Gel/Mount and a coverslip were applied. Sections without primary antibodies applied or those receiving the primary antibodies preneutralized with fivefold excess of corresponding blocking peptides for 2 h were used as negative controls. The staining was evaluated under an epifluorescent microscope (Eclipse 400; Nikon, Inc., Melville, NY) and photographed with a digital camera (model DMX 1200; Nikon).

### Western blot analysis

To confirm the presence of NTF proteins in limbal and corneal epithelia, western blot analysis was performed using NTF-specific antibodies. Fresh normal human limbal and corneal epithelia were lysed in RIPA buffer (50 mM Tris-HCl, 150 mM NaCl, 1% NP-40, 0.5% sodium deoxycholate, 2 mM sodium fluoride, 2 mM EDTA, 0.1% SDS, and an EDTA-free protease inhibitor cocktail tablet), and protein concentration was determined using the Pierce BCA method, calibrating against standards of known BSA concentration. Western blot analysis was performed using a previously described method [32] with modifications. The epithelial extracts (30 μg total protein/lane) were mixed with 6X SDS reducing sample buffer and boiled for 5 min before loading. Proteins were separated by SDS PAGE and transferred electronically to PVDF membranes. The membranes were blocked with 5% non-fat milk in Tris buffered saline with 0.1% Tween20 (TTBS) for 1 h at RT and then incubated for 2 h at RT with pAbs to NGF (1:150), GFRα-1 (1:100), or GDNF (1:200) or with mAb to p75NTR (1:500). The membranes were washed with TTBS and incubated for 1 h at RT with horseradish peroxidase-conjugated goat anti-rabbit IgG (1:3000). After washing the membranes, the signals were detected with an ECL immunodetection kit and then exposed to X-ray film (Eastman Kodak, Rochester, NY) from 30 s to 3 min. α-Actin was used as an internal standard.

## Results

### Immunolocalization of neurotrophic factors and their receptors in human corneal and limbal tissues

**Figure 1:**
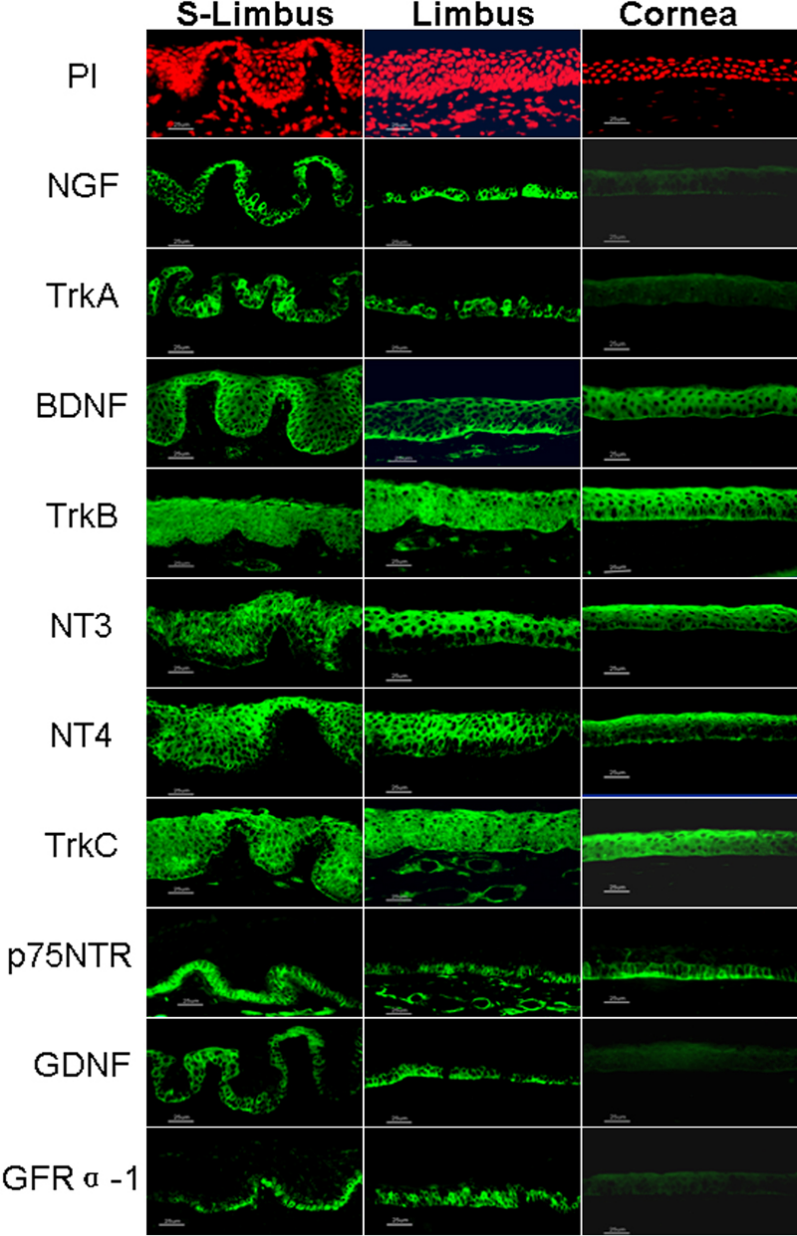
Expression of neurotrophic factors and corresponding receptors in human corneal tissue

Immunofluorescent staining was used to spatially localize six members of three NTF families and their six corresponding receptors in corneal and limbal tissues. Staining was evaluated in sections cut in two different orientations, cross-sectional and meridional, shown with PI counterstaining in the top panel of Figure 1. The horizontal cross-section cut through the superior limbus (S-Limbus) showed the papilla-like limbal epithelial columns and interspersed blood vessels, nerves, and connective tissue between the epithelial columns of the palisades of Vogt environment. The meridional sections cut from the limbus through the central cornea displayed a traditional limbus (Limbus) with about 8–10 layers of epithelium and vessel and nerve-enriched stroma as well as the central cornea (Cornea) with about five layers of corneal epithelium, Bowman's layer, and avascular stroma. Patterned immunolocalization of these NTFs and their receptors were characterized in these tissues.

Representative images showing the immunofluorescent staining of NGF, TrkA, BDNF, TrkB, NT-3, NT-4, TrkC, p75NTR, GDNF, and GFRα-1, (green color) in frozen sections of human corneoscleral tissues: tangential cross sections cut through the superior limbus (S-Limbus, left column), radial meridional sections cut through limbus (Limbus, central column), and central cornea (Cornea, right column). Propidium iodide (PI) was used as nuclear counterstaining (red). The images in the top panel are matched to the images in the NGF panel as examples; other PI images matched to each NTF staining are not shown. Magnification: x400 (bar=25 μm).

### Neurotrophins and their receptors

As shown in the second panel of Figure 1, NGF immunoreactivity was found to be exclusively localized to a subset of cells in the basal epithelial layer of the human limbus, but it was totally negative in the entire cornea and suprabasal layers of the limbal epithelium. Clusters of NGF immuno-positive basal cells were seen to be interspersed between negative cell patches. The expression pattern of NGF receptor, TrkA, noted in these sections was consistent with the previous reports [28,33]. TrkA was confined primarily to the basal layer of the limbal epithelium with clusters of brightly positive cells interspersed between a few negative cells. TrkA expression also extended to some suprabasal epithelial cells in the layer just above the basal limbal epithelial cells. A few small clusters of TrkA positive cells were seen in the basal epithelial layer of the peripheral cornea. Similar to NGF, no TrkA immunoreactivity was detected in the corneal and limbal stroma (Figure 1, third panel). This specific immunoreactivity to NGF and TrkA was abolished in the negative controls where the antibodies were neutralized by their corresponding blocking peptides.

In contrast to NGF, BDNF immunostaining (Figure 1, fourth panel) was noted in all epithelial layers of the cornea and limbus with the strongest staining in the basal layer of limbal epithelium compared to equal staining intensity throughout all layers of the corneal epithelium. BDNF was also widely expressed by limbal stromal cells including vessel endothelial cells, nerves, and fibroblasts. TrkB serves as a high affinity receptor, primarily for BDNF and NT4. The TrkB antibody evenly stained almost all layers of the corneal and limbal epithelium. Similar to BDNF, TrkB was also expressed by most stromal cells in the limbal region (Figure 1, fifth panel).

NT-3 staining was noted in the uppermost layers of the corneal and limbal epithelia. The basal cells were almost negative, especially in the limbus (Figure 1, sixth panel). The expression pattern of NT-4 was similar to NT-3 in the human corneal and limbal epithelia (Figure 1, seventh panel). Neither NT-3 nor NT-4 were detected in the stroma of these tissues. Similar to TrkB, TrkC staining was evenly distributed throughout the entire corneal and limbal epithelia and their stroma cells (Figure 1, eighth panel).

p75NTR, a low affinity receptor for all neurotrophins, was found to uniquely stain the basal layer and immediate suprabasal layer of the corneal and limbal epithelia, including the central corneal epithelium. The suprabasal epithelial layers in the cornea and limbus were negative. Stroma cells including vessel endothelial cells, nerves, and fibroblasts in the limbal area were positively stained (Figure 1, ninth panel).

### Glial cell-derived neurotrophic factor family

Interestingly, both GDNF and its specific receptor GFRα-1 were exclusively immunolocalized to the basal layer of the limbal epithelium. The entire corneal epithelium, suprabasal limbal epithelium, and stromal cells were totally negative (Figure 1, bottom two panels). This expression pattern was similar to NGF.

### Ciliary neurotrophic factor family

No specific immunoreactivity to CNTF and its receptor, CNTFRα, was detected in the entire cornea and limbus (data not shown).

### Western blot analysis of neurotrophic factors and their receptors in human corneal and limbal epithelia

**Figure 2:**
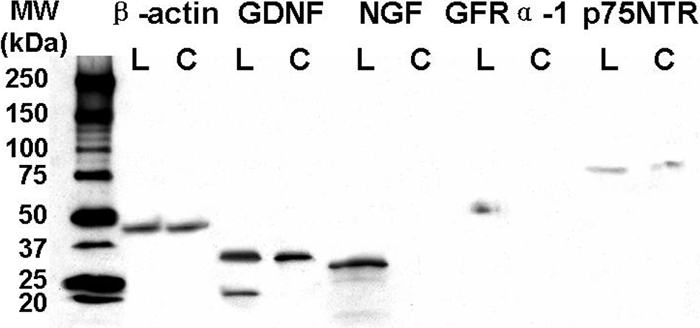
Western blot analysis

The presence of NGF, GDNF, and their receptors, p75NTR and GFRα-1, in human corneal and limbal epithelial tissues were confirmed by western blot analysis using β-actin (45 kDa) as a positive control (Figure 2). NTFs can be detected at multiple molecular weights because they are present in tissues as pro-forms, mature forms, dimers, and glycosylated forms at multiple sites [34-36]. Pro-NGF has a molecular weight of 34 kDa whereas the mature form has a molecular weight of 14 kDa. In limbal epithelial tissue, the major band detected was pro-NGF, around 30 kDa, and two additional bands of weaker intensity at 25 and 14 kDa (mature NGF) were also noted. In contrast, NGF in any form was not detected in the corneal epithelium. The reported molecular weight of GDNF is 21 kDa [37] and its glycosylated, disulfide-bonded homodimer protein has been noted to be about 35 kDa [36]. A 35 kDa band of glycosylated GDNF was detected in both limbal and corneal epithelia while the 21 kDa band was only detected in the limbal epithelium. A 53 kDa GFRα-1 band [37] was detected only in limbal epithelium. The 75 kDa p75NTR protein was expressed in both limbal and corneal epithelia.

Western blot analysis of GDNF, NGF, GFRα-1, and p75NTR in human corneal (C) and limbal (L) epithelial tissues with β-actin (45 kDa) as a control. NGF (a major band at approximately 30 kDa, and two weaker bands at 25 and 14 kDa) and GFRα-1 (53 kDa) were detected only in the limbal epithelium and not in the cornea. A 35 kDa band of glycosylated GDNF was detected in both limbal and corneal epithelia while an additional 21 kDa band was only detected in limbal epithelium. The p75NTR protein (75 kDa) was detected in both limbal and corneal epithelia.

## Discussion

Although neurotrophic factors are defined as polypeptides that maintain neuronal cells, they have been reported to possess a range of functions in promoting survival and self renewal of stem cells outside the nervous system (see review articles [15,17]). While previous studies have reported that several NTFs and their receptors were expressed by corneal epithelial and stroma cells and that NGF and GDNF stimulated corneal epithelial proliferation in culture [25-28], the spatial localization and biologic role of these neurotrophic factors on the ocular surface has not been well elucidated. Our study provides a comprehensive view of the unique expression patterns of the most important members of the NTF family and their receptors in human cornea and limbus although these immunostaining patterns alone can not convincingly extrapolate to what may actually happen in vivo.

### Three patterns of neurotrophic factor expression are potentially involved in epithelial-mesenchymal interactions on the ocular surface

It was well known that epithelial-mesenchymal interactions play a vital role in embryonic development, postnatal morphogenesis, wound healing, and tumor metastasis as well as in stem cell maintenance in a variety of tissues (see review articles [38-40]). We have previously reported three expression patterns of cytokines potentially involved in epithelial-fibroblast interactions on the human ocular surface [9]: (1) Type I (epithelial type): Transforming growth factor (TGF)-α, interleukin (IL)-1β, and platelet-derived growth factor (PDGF)-B were expressed exclusively by epithelial cells, but their respective receptors, epidermal growth factor receptor (EGFR), IL-1R, and PDGFR-β, were predominantly expressed by fibroblasts; (2) Type II (reciprocal type): Insulin-like growth factor (IGF)-I, TGF-β1, -β2, leukemia inhibitory factor (LIF), basic fibroblast growth factor (bFGF), and their receptors were expressed by both epithelium and fibroblasts; and (3) Type III (fibroblast type): Keratinocyte growth factor (KGF) and hepatocyte growth factor (HGF) were expressed exclusively by fibroblasts, and their respective receptors, KGFR and c-met, were predominantly expressed by epithelial cells. Based on their differential immunolocalization, the NTFs and receptors can also be categorized into three expression patterns on the ocular surface. Two NTFs, NGF and GDNF, and their corresponding high affinity receptors, TrkA and GFRα-1, were exclusively expressed by corneal, limbal, and conjunctival (data not shown) epithelia, but they were not detected in stromal mesenchymal cells in these tissues. While not specifically evaluated, these finding suggest that NGF and GDNF may play a major role in corneal epithelial maintenance and regeneration although NGF may also have effects on stromal cells in these tissues through the common neurotrophin receptor, p75NTR. Thus, these two NTFs may represent a unique “epithelial type” of NTF in non-neuron tissues on the ocular surface. NT-3 and NT-4 were also expressed only by ocular surface epithelial cells, but they may have effects on epithelial and stromal cells both of which expressed their receptors, TrKB and TrkC. Therefore, NT-3 and NT-4 may represent a “paracrine type” of NTF, modulating both epithelial and mesenchymal cells. A third type of expression pattern was noted for BDNF and three neurotrophin receptors, TrKB, TrkC, and p75NTR, which were expressed by both epithelial and stromal cells, suggesting that BDNF may represent a “reciprocal type” of NTF that mediates epithelial-mesenchymal interactions on the ocular surface. Our characterization of these three patterns of NTF expression may facilitate future investigations to specifically define the roles of NTFs in maintaining ocular surface homeostasis and in corneal wound healing, epithelial regeneration, and stem cell survival.

### Expression patterns of neurotrophic factors and their receptors in human limbal basal epithelium

Since the corneal epithelial stem cells were found to reside at the limbus over two decades ago [41], several markers for these stem cells have been proposed though no definitive molecular markers for these stem cells have been identified to date [42-45]. We have characterized a unique phenotype of stem cell-enriched human limbal epithelial basal cells and proposed that corneal epithelial stem cells, also referred to as limbal stem cells, are small primitive cells expressing three patterns of molecular markers [30]: (1) exclusively positive for p63, ABCG2, and integrin α9 by a subset of basal cells; (2) relatively higher expression of integrin α1, EGFR, K19, and α-enolase by most basal cells, and (3) lack of expression of nestin, E-cadherin, connexin 43, involucrin, K3, and K12. N-cadherin has also been demonstrated to be exclusively expressed by certain limbal basal cells [46]. TrkA, a high affinity receptor for NGF, was found to be expressed by limbal basal epithelial cells and was proposed as a potential limbal stem cell marker [28,45]. The present study further investigated the expression and localization of NTF families in the limbal basal epithelial layer in situ.

Touhami et al. [28] examined the immunoreactivity of NGF and its specific receptors in human limbal epithelium and reported that no staining for NGF was observed in the entire cornea and p75NTR staining was negative in limbal basal epithelium. Using different antibodies to NGF and p75NTR, results of our immunofluorescent staining and western blot studies showed that NGF was uniquely expressed in the human limbal epithelium with its two corresponding receptors, high-affinity TrkA and low-affinity p75NTR. Interestingly, NGF and GDNF were only expressed by epithelial cells on the ocular surface, and immunofluorescent staining showed that they were exclusively confined to the limbal basal epithelial layer where stem cells reside. Clusters of NGF and GDNF positive cells were interspersed between negative basal cells in the limbal palisades (Figure 1, S-Limbus). Western blot showed that the expression of non-glycosylated GDNF was detected only in the limbal epithelium. Their corresponding high affinity receptors, TrkA and GFRα-1, were also localized primarily to the basal limbal epithelial cells, sharing a similar expression pattern to their ligands (Figure 2). Western blot also supported the finding that GFRα-1 protein was only produced by the limbal epithelium. In the limbal epithelium, expression of the neurotrophin, BDNF, was stronger in the basal cells than in the suprabasal cells, similar to the expression pattern that has been reported for integrin α1 and EGFR [30]. NT-3 and NT-4 were expressed by the majority of corneal and limbal epithelia, but the basal epithelial layer of these tissues was negative. Thus, three expression patterns of these NTFs by limbal basal cells were characterized: (1) exclusively positive for NGF, GDNF, and their receptors, TrkA and GFRα-1, (2) relatively higher level for BDNF, and (3) negative for NT-3 and NT-4. These findings suggest that NTFs are potential new markers for limbal basal cells (Table 1), thus expanding the previously proposed limbal stem cell phenotype [30].

**Table 1:** Three expression patterns of limbal basal cell markers

		Limbal epithelium		Corneal epithelium	
Pattern	Markers	Basal	Suprabasal	Basal	Suprabasal
I	p63	+++	-	-	-
	ABCG2	+++	-	-	-
	Integrin α9	+++	-	-	-
	N-cadherin	+++	-	-	-
	NGF*	+++	-	-	-
	TrkA*	+++	-	-	-
	GDNF*	+++	-	-	-
	GFRα-1*	+++	-	-	-
II	Integrin β1	+++	+	+++	++
	EGFR	+++	+	+++	++
	K19	+++	+	+++	+++
	α-enolase	+++	+	++	+
	BDNF*	+++	++	++	++
III	Nestin	-	+++	+	+++
	E-cadherin	-	+++	+	+++
	NT-3*	-	+++	+	+++
	NT-4*	-	+++	+	+++
	Connexin 43	-	+++	+	+++
	Involucrin	-	+++	+	+++
	K3	-	+++	+++	+++

The evaluation was based on immunofluorescent staining. In the table, -, undetectable; +, weakly positive; ++, moderately positive; +++, strongly positive. The asterisk indicates neurotrophic factors or their receptors.

In addition, the neurotrophin low-affinity receptor, p75NTR, displayed a unique expression pattern with its immunoreactivity localized to the basal layer and its closest suprabasal layer throughout the entire limbal and corneal epithelia including the central cornea. Western blot supported this finding. The expression pattern for p75NTR was different from all proposed stem cell associated markers thus far, suggesting that p75NTR has a similar distribution pattern to the transient amplifying cells (TACs).

In conclusion, our findings revealed patterned expression of NTFs and their receptors on the human ocular surface, suggesting that NGF, GDNF, GFRα-1, TrkA, and BDNF may serve as new limbal basal cell markers for the corneal epithelial stem cell phenotype. Further studies are needed to explore the functional role of different NTFs in maintaining the corneal epithelial stem cells in the limbal niche.
